# Develop an indirect ELISA utilizing gD protein to detect antibodies against bovine herpesvirus type 1

**DOI:** 10.3389/fcimb.2025.1591304

**Published:** 2025-05-08

**Authors:** Qiang Liu, Ruijin Liang, Xiaoxia Niu, Lingling Jiang, Gang Zhang, Pu Wang, Sinong Zhang, Weifeng Gao, Yujiong Wang, Huichen Guo, Yong Li

**Affiliations:** ^1^ Key Lab of Ministry of Education for Protection and Utilization of Special Biological Resources in Western China, School of Life Sciences, Ningxia University, Yinchuan, China; ^2^ Queen’s University Belfast Joint Institute, China Medical University, Shenyang, China; ^3^ State Key Laboratory of Veterinary Etiological Biology, College of Veterinary Medicine, Lanzhou University, Lanzhou Veterinary Research Institute, Chinese Academy of Agricultural Sciences, Lanzhou, China

**Keywords:** bovine herpesvirus type 1, gD protein, prokaryotic expression, indirect ELISA, antibody detection

## Abstract

Bovine herpesvirus type 1 (BHV-1) is a highly contagious DNA virus that causes a variety of diseases affecting the reproductive and respiratory tracts. These diseases can reduce the health and production performance of cattle, causing significant economic losses in the cattle industry. The current ELISA kits used to detect BHV-1 have long lead times and are expensive, and are not suitable for bulk testing on large farms. therefore, there is an urgent need to develop a rapid and cost-effective alternative to the BHV-1 test. In this study, recombinant gD protein was expressed by prokaryotic system, and then used as antigen to immunize New Zealand white rabbits to obtain polyclonal antibodies (pAb). An indirect enzyme-linked immunosorbent assay (iELISA) based on gD protein was established for the detection of BHV-1 antibodies in clinical samples. The optimal cutoff value was determined to be 0.6185 using 60 clinical serum samples. This method had no cross-reaction with other common bovine viruses. The developed iELISA method and commercially available kits were used to detect 60 bovine serum samples, with a concordance rate of 93.3%. In summary, we established a rapid and reliable iELISA method based on gD protein, which is suitable for epidemio-logical monitoring of BHV-1.

## 1 Introduction

BHV-1 is a virus responsible for infectious bovine rhinotracheitis in cattle populations, primarily manifesting as abortion, infertility, and endometritis in female cattle (Wathes et al., 2020). Despite a modest death rate post-infection, BHV-1 can induce prolonged latent infections, significantly reducing the economic efficiency of cattle farms and causing substantial economic losses globally (Ostler and Jones, 2023). The virus has been listed by the World Organization for Animal Health (WOAH) as a notifiable animal pathogen (Biswas et al., 2013). BHV-1 was first discovered in dairy cows in California, USA in 1953 and became widespread in many European countries in the 1960s (Turin et al., 1999), and was first isolated from imported dairy cattle in China in 1840, subsequently becoming prevalent in various regions (Guo et al., 2022). Effective control of BHV-1 relies on the precision of detection methods, with proteins such as gB and gD being targeted for the development of diagnostic reagents (Werid et al., 2023).

BHV-1 is an enveloped, double-stranded DNA virus with a genome size of approximately 136 kb, encoding a total of 73 proteins, which includes 33 structural proteins and up to 15 non-structural proteins (Romera et al., 2022). Viral glycoproteins play a crucial role in the interaction between the virus and host cells, involved in processes such as attachment, invasion, assembly, and release (Marawan et al., 2021). At least six glycoproteins (gB, gC, gD, gH, gK, gL) have been demonstrated essential for the invasion process (Hidayati et al., 2018). The gD protein serves as a principal structural component of BHV-1 and functions as a typical viral ligand for the entry receptor. The entry of the virus into cells is contingent upon the binding of gD to receptors on the cell surface (Yue et al., 2020). Research demonstrates that the gD protein typically elicits a more robust humoral and cell-mediated immune response in comparison to other proteins. Subunit and DNA vaccines based on this glycoprotein have been developed and evaluated for their immunogenic potential (Krishnagopal and van Drunen Littel-van den Hurk, 2024). The gD protein is regarded as an ideal antigen for diagnosing BHV-1 infection and evaluating vaccine immunogenicity, owing to its immunodominance and conserved sequence.

Timely and precise diagnostic practices are essential for the implementing proactive and effective containment strategies. ELISA stands as a commonly used serological assay that detects presence of antibodies or antigens (Zhang et al., 2024). The high sensitivity, specificity, and simplicity of the method make it the preferred choice for WOAH. In recent decades, various ELISA variants, such as iELISA, competitive ELISA, and sandwich ELISA, have been extensively employed in the detection of animal pathogens, these assays primarily focus on the recombinant structural proteins of viruses (Ahirwar et al., 2022).

In this study, we used a prokaryotic system to express recombinant gD protein, prepared rabbit polyclonal antibodies against gD protein, and established a simple and rapid iELISA method for detecting BHV-1 antibodies based on gD protein, which will be helpful for large-scale clinical sample testing and epidemiological surveys in cattle farms.

## 2 Materials and methods

### 2.1 Strains, animals and samples

*Escherichia* coli (*E. coli*) DH5α and BL21 (DE3) were acquired from Sangon Biotech (Shanghai, China) Male New Zealand White rabbits, approximately two months old and weighing 2~2.5 kg, were purchased and raised at the Animal Center of Ningxia Medical University (Yinchuan, China). The positive serum samples of cattle for BHV-1, bovine coronavirus (BCoV), bovine viral diarrhea virus (BVDV), bovine rotavirus (BRV), and bovine respiratory syncytial virus (BRSV) were stored in the laboratory.

### 2.2 Construction of recombinant plasmid for gD protein

The gD gene exhibits a degree of conservation, with sequences from 24 BHV-1 strains sourced from China, Inner Mongolia, India, the United States, and France obtained from NCBI for homology analysis. The results showed that 24 strains exhibited homology between 97% to 100%. Based on the sequencing results of BHV-1 from clinical samples in our laboratory, The gD protein sequence with GenBank accession number QBI59528.1 was ultimately selected. We used bioinformatics tools to predict and analyze the gD protein, using TMHMMServer2.0 to predict the transmembrane region of the gD protein sequence, SignalP6.0 to analyze the position of the signal peptide sequence, ProtScale to predict the hydrophobicity of the gD protein, and IEDB online website to predict B cell epitopes. The signal peptide and transmembrane region were removed from the gD sequence, a 6×His tag was added before the stop codon, and restriction sites *Eco*RI and *Hin*dIII were introduced upstream and downstream of the gene, respectively. The gene was subsequently cloned into the corresponding restriction sites of the pET-32a (+) vector, and the recombinant plasmid was named pET32a-gD. Codon optimization and gene synthesis were performed by GenScript Biotech (Nanjing, China), with E. coli as the host. The synthesized recombinant plasmid was transformed into BL21(DE3) and incubated overnight at 37°C on agar plates with 100 µg/mL ampicillin (Du et al., 2022). Single colonies were picked and cultured in LB liquid medium for expansion, and the recombinant plasmid was extracted and validated through restriction digestion and DNA sequencing (Liu et al., 2024).

### 2.3 Expression and purification of recombinant gD protein

The recombinant BL21(DE3) strain, induced with 0.5 mM IPTG, was expressed at 37°C for 6 h. And as mentioned earlier (Shen et al., 2023), the expression of the protein was analyzed by SDS-PAGE. The protein was further purified using a gravity column filled with Ni Focurose HP (IMAC) resin (VDOBIOTECH, Suzhou, China).

### 2.4 Western blotting (WB)

As previously described (Wang et al., 2022), the purified gD protein was separated by SDS-PAGE and transferred to a PVDF membrane through wet transfer. The membrane was blocked with TBS containing 5% skim milk powder (SMP) for 2 h. It was then incubated overnight with a rabbit Anti-6×His tag monoclonal antibody (Abcam, Shanghai, China), followed by a 1 h incubation with HRP-conjugated Affinipure Goat Anti-Rabbit IgG(H+L) (Proteintech, Wuhan, China). The protein bands were visualized using a 1:1 mixture of ECL Plus ultra-sensitive luminescent liquid A and B (Solarbio, Beijing, China), images were acquired using a chemiluminometer (GE, Amersham Imager 600).

### 2.5 Rabbit immunization and preparation of rabbit pAb against gD protein

Endotoxin-free recombinant gD protein was mixed in a 1:1 ratio with Freund’s Complete Adjuvant (Sigma, Shanghai, China) and administered through multiple subcutaneous injections in the dorsal region of rabbits, with a negative control group established concurrently. The initial and fourth immunizations were utilized 1 mg of the protein to enhance the immune response (Greenfield, 2020). While the second and third immunizations used 0.5 mg of the protein. At the eighth weeks post-immunization, the immunized rabbits were anesthetized, and blood was collected via cardiac puncture for serum separation. The immunogenicity of the antiserum was evaluated using iELISA with the purified gD protein as the coated antigen (Liu et al., 2017).

### 2.6 Purification and characterization of pAb

Rabbit antiserum was purified using gravity columns filled with Protein A Focurose HR resin (Huiyan Biological, Wuhan, China), with all operations performed at 4°C (Sompunga et al., 2019). Briefly, the antiserum was mixed with an equilibration buffer in 1:2 ratio, filtered through a 0.22 μm filter, and then loaded onto the purification column. After a 1 h incubation, the antibodies were eluted twice with 3 mL of Glycine buffer (pH 3.0) and neutralized with 80 μL of Tris-HCl (pH 8.8) (Sensi and Goebel, 2022). The antibodies were preserved in neutral pH conditions. Antibody samples were prepared for electrophoresis and analyzed using SDS-PAGE. The purified pAb were employed as primary antibodies to determine their specific recognition of the recombinant gD protein by WB.

### 2.7 Indirect immunofluorescence assay

Indirect immunofluorescence assay (IFA) was employed to detect the interaction between rabbit pAb and BHV-1 (Li et al., 2022). Well-growing MDBK cells were seeded at a density of 1×106 cells/well in a six-well plate containing a cell slide. The next day, when the cells reached about 90% confluence, 200 μL of BHV-1 after repeated freezing and thawing was added to each well, and new complete medium was replaced after incubation for 2 h. After 48 h, the cell lesions were observed under a microscope for IFA detection. The specific steps are: discard the culture medium, wash the slides 3 times with PBS; fix with 500 μL 4% paraformaldehyde at room temperature for 10 min, wash 5 times with PBST, 3 min each time; add 0.2% Triton X-100, incubate at room temperature for 15 min to permeabilize the cells, wash 3 times with PBST, 5 min each time; add 5% BSA, evenly cover the cell sheet, block at room temperature for 1 h, wash 3 times with PBST, 5 min each time; The purified rabbit pAb were diluted 1:2000 as the primary antibody, incubate at 37°C for 2 h, and CoraLite594 conjugated Goat Anti-Rabbit IgG(H+L) (Proteintech, Wuhan, China) was diluted 1:1000 as the secondary antibody for incubation, incubate in dark for 1 h, wash three times with PBST, 5 min each time, and add anti-fluorescence quenching sealing solution containing DAPI to seal the slides (Beyotime, Shanghai, China). Laser confocal microscope (OLYMPUS, LEXT™OLS5100) was used for observation and photography (Gagliardi et al., 2022).

### 2.8 Optimization of iELISA conditions based on gD protein

The optimal antigen coated concentration and serum dilution for iELISA were studied through a checkerboard titration method (Jin et al., 2023). Recombinant gD protein was diluted with carbonate buffer to concentrations of 8.0, 4.0, 2.0, 1.0, 0.5, and 0.25 μg/mL and coated 100 μL overnight at 4°C on an ELISA plate. Blocking agents (1% BSA, 3% BSA, and 5% SMP) were added to the wells at a volume of 150 μL and incubated at 37°C for 2 h. After washing three times and patting dry, diluted rabbit pAb (1:100, 1:200, 1:400, 1:800) and corresponding negative serum were added to the wells and incubated at 37°C for 1 h. Wash three times and pat dry, incubate with 100 μL HRP-conjugated affinipure goat anti-rabbit IgG at dilutions of 1:5000, 1:8000, and 1:12000 for 30, 45, and 60 minutes at 37°C. Wash three times and pat dry, incubate wells with 100 μL 3,3’,5,5’-tetramethylbenzidine (TMB) substrate for 10, 15, and 20 minutes at 37 °C in the dark. Terminate the reaction with 50 μL 2 M H_2_SO_4_. All data were repeated three times independently, and the absorbance values at 450 nm were measured using a multi-function ELISA reader (PerkinElmer, EnSpire). A positive reaction is usually defined as an absorbance ratio greater than 2.1 between the positive and negative controls.

### 2.9 Determination of the cutoff Value

Under the optimal conditions mentioned above, iELISA was employed for measuring the OD450 values of 30 negative and 30 positive bovine clinical serum samples, with each serum samples tested in triplicate. All bovine serum samples were differentiated and confirmed in our laboratory using classical PCR detection techniques and DNA sequencing. Receiver operating characteristic (ROC) curve analysis was employed to analyze the iELISA results of positive and negative samples to determine the cutoff value, maximizing the diagnostic specificity and sensitivity of the detection method. The area under the curve (AUC) within the 95% confidence interval derived from the ROC curve analysis reflects the accuracy of the detection method, the closer the AUC is to 1, the more accurate the method. The Youden index was used to find the appropriate critical value and evaluate the overall performance of iELISA.

### 2.10 Analysis of specificity and sensitivity of iELISA

To evaluate the diagnostic sensitivity of the method, the rabbit polyclonal serum was diluted from 2^12^ to 2^19^. When the OD450 measurement value exceeded the critical value, it was positive, and the highest dilution factor of the positive result was the detection limit of iELISA. Three independent tests were performed for each group to ensure the reproducibility of the data.

### 2.11 Reproducibility analysis the accuracy of the established iELISA

The iELISA method is assessed through intra-batch and inter-batch variability. Under optimal conditions, iELISA is employed to analyze six positive serum samples and two serum negative samples, with three repetitions for each dataset, calculating the mean, standard deviation, and coefficient of variation (CV) of each sample. The degree of variation of iELISA is characterized by the coefficient of variation, thereby evaluating the accuracy of the method.

### 2.12 Comparison of iELISA and commercially ELISA kits

A total of 60 clinical bovine serum samples from cattle farms in Gansu Province were tested using the developed iELISA method and a commercial antibody detection iELISA kit (Keshun Biotechnology, Shanghai, China). The relative sensitivity and specificity of the two methods were analyzed through the experimental results, and the coincidence rate of the two methods was calculated (Lalkhen and McCluskey, 2008).

## 3 Results

### 3.1 Construction and characterization of pET32a-gD recombinant vector

Predictive analysis of the gD protein sequence using bioinformatics software showed that the transmembrane region of the hydrophobic gD protein was located at amino acid residues 365~387 (**Supplementary Figures S1A, C**); the signal peptide was situated between amino acid residues 1~18 (**Supplementary Figure S1B**), B-cell epitope prediction revealed multiple antigenic epitopes, mainly concentrated between amino acid residues 200~415, with the majority distributed in the extracellular region (**Supplementary Figure S1D**), which can stimulate the production of humoral immunity, demonstrating strong immunogenicity and suitability for the development of target antigens for pathogen detection.

**Figure 1:**
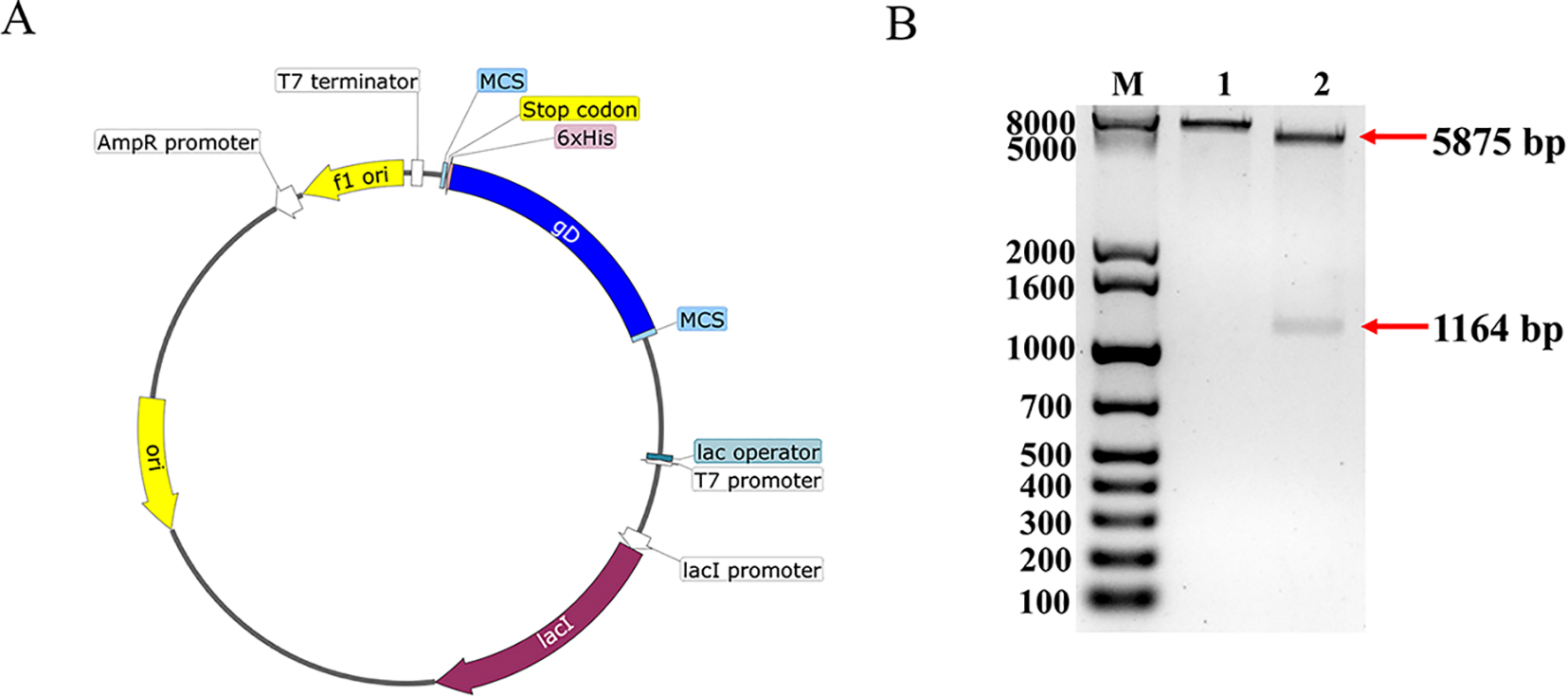
Construction and characterization of BHV-1 gD protein vector. **(A)** Construction mapping of pET32a-gD prokaryotic expression vector. **(B)** Enzymatic characterization of pET32a-gD prokaryotic expression vector. Lane M, 1kb Plus DNA Marker; lane 1, *Hin*dIII single digestion; lane 2, *Eco*RI and *Hin*dIII double digestion.

Following removing the signal peptide and transmembrane region, the gD sequence was optimized for codon preference, mRNA secondary structure, GC content and repetitive sequences based on E. coli host, resulting in the optimization of the GC content to 68.47% and an increase in the codon fitness index from 0.65 to 0.75. The construction was performed according to the predicted information, and the recombinant plasmid pET32a-gD was synthesized (**Figure 1A**), after double enzyme digestion, the recombinant plasmid obtained a 5881 bp band and a 1158 bp target band (**Figure 1B**), which was consistent with the expected results, and the DNA sequencing comparison results were also completely correct. These results showed that the pET32a-gD recombinant plasmid was successfully constructed.

### 3.2 Recombinant gD protein expression and purification validation

**Figure 2:**
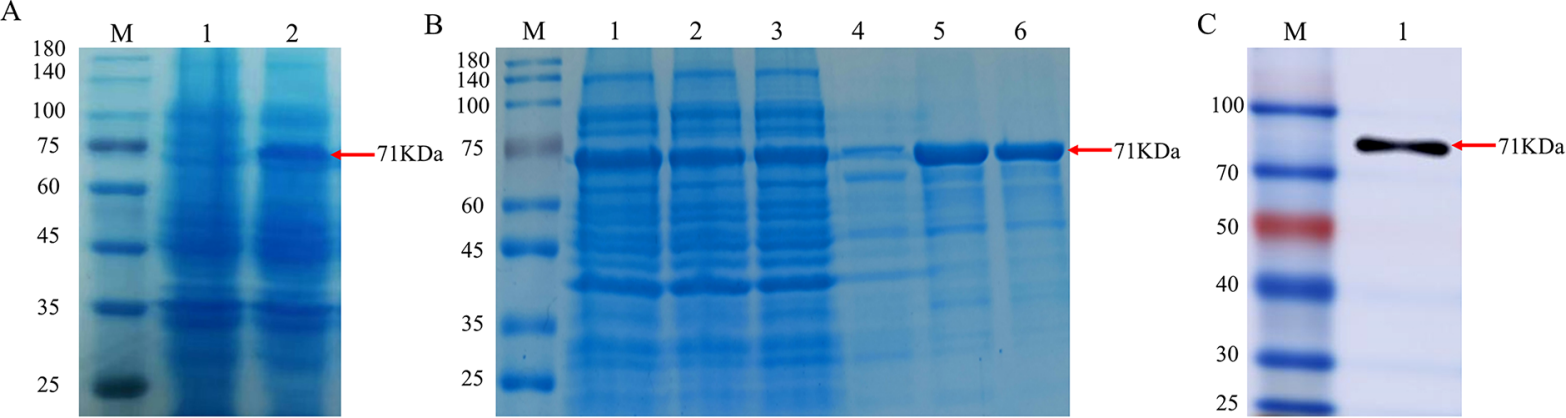
Identification of recombinant gD protein. **(A)** SDS-PAGE analysis of recombinant BHV-1 gD protein expression. Lane M, protein marker; lane 1, whole bacterial protein after transformation with pET32a plasmid; lane 2, whole bacterial protein after transformation with pET32a-gD plasmid. **(B)** SDS-PAGE analysis of purified protein. Lane M, protein marker; lane 1, whole bacterial protein; lane 2, supernatant of bacterial lysate; lane 3, flow-through; lane 4, 50 mM imidazole elution; lane 5, 250 mM imidazole elution; lane 6, 500 mM imidazole elution. **(C)** WB identification of recombinant BHV-1 gD protein using rabbit anti-6×His monoclonal antibody. Lane M, protein marker; lane 1, purified BHV-1 gD protein.

Induction was carried out using 0.5 mM IPTG, and the positive clone strain BL21(DE3) was incubated at 37°C for 6 h. The results of SDS-PAGE demonstrated differential banding at approximately 71 KDa compared to the blank control group (**Figure 2A**), indicating that the gD protein was induced to express successfully, followed by affinity chromatography purification of the sonicated supernatant, and the purified results were analyzed by SDS-PAGE, with specific gD proteins eluting at 250 mM and 500 mM imidazole concentrations (**Figure 2B**). A single band from WB analysis again confirmed that the successful expression and purification of the gD protein (**Figure 2C**).

### 3.3 Preparation and characterization of rabbit pAb

Using recombinant gD protein as coating antigen, the titer of antiserum was 4.096×105 as determined by iELISA (**Table 1**).

**Table 1:** Determination of rabbit antiserum potency of BHV-1 gD protein.

Serum dilution (×100)	Positive (P) OD450	Negative (N) OD450	P/N
1	4.51	0.30	15.04
2	4.32	0.29	14.88
4	4.46	0.28	15.94
8	4.31	0.28	15.39
16	4.32	0.21	20.55
32	4.33	0.27	16.05
64	3.93	0.22	17.86
128	3.35	0.21	15.95
256	2.84	0.22	12.90
512	1.57	0.19	8.25
1024	0.80	0.17	4.69
2048	0.62	0.14	4.45
4096	0.29	0.13	2.22
8192	0.18	0.14	1.26

**Figure 3:**
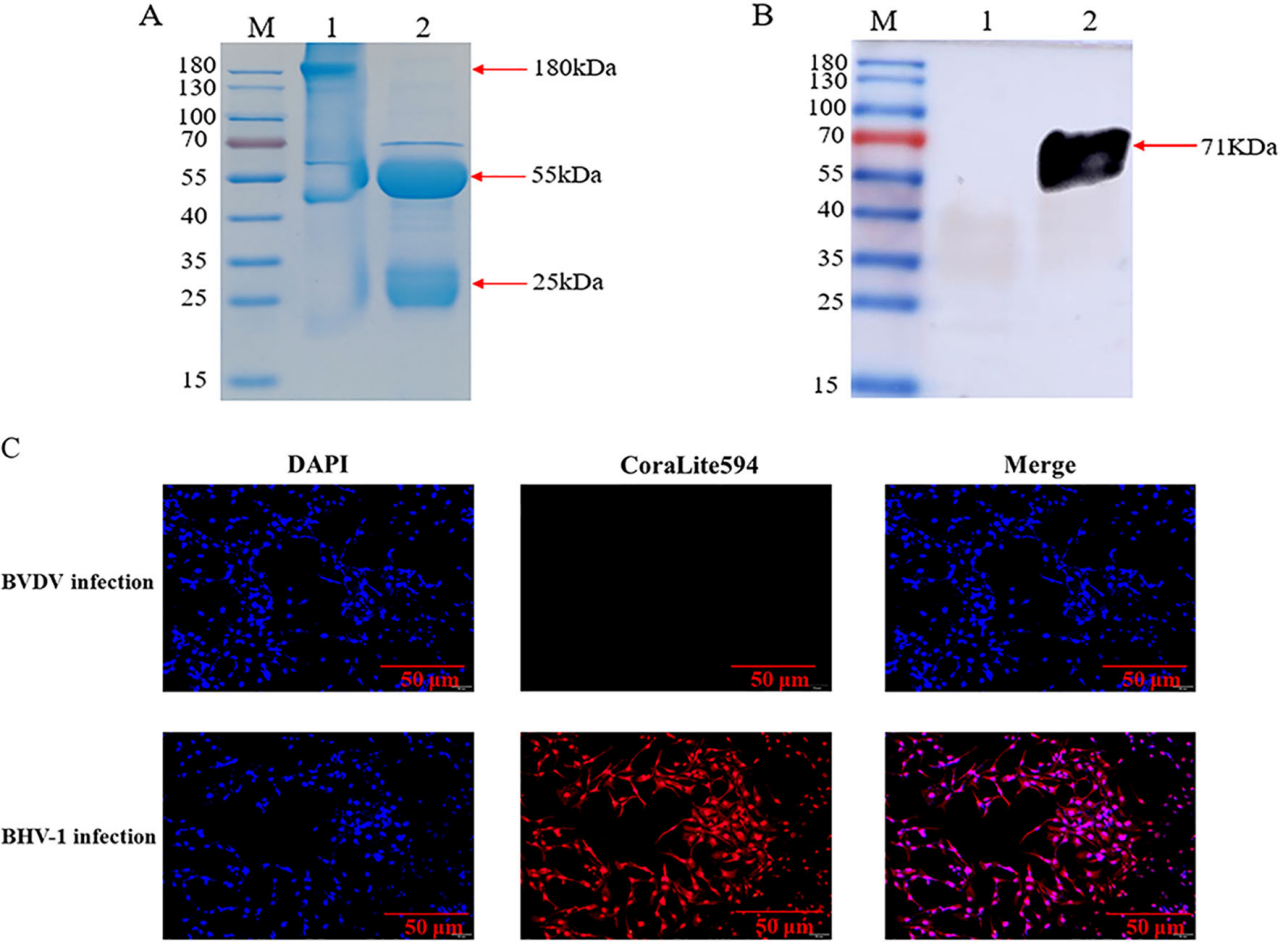
Characterization of rabbit pAb against gD protein. **(A)** SDS-PAGE analysis of purified rabbit pAb. Lane M, protein marker; lane 1, purified non-reduced rabbit pAb; lane 2, purified reduced rabbit pAb. **(B)** WB characterization of recombinant gD protein using purified rabbit pAb as a primary antibody. Lane M, protein marker; lane 1, bacterial protein after empty plasmid pET32a was transfected into BL21(DE3); lane 2, recombinant gD protein. **(C)** IFA analysis of rabbit pAb targeting BHV-1.

The centrifuged immunized rabbit serum was purified, and the SDS-PAGE results of the purified reduced and non-reduced rabbit pAb demonstrated that the rabbit pAb was structurally intact and had a natural conformation with an approximate size of 180 KDa (**Figure 3A**), Additionally, WB analysis revealed that the purified rabbit pAb had good immune reactivity (**Figure 3B**). We infected MDBK cells with BHV-1 and BVDV, respectively, and assessed the specificity of rabbit pAb for BHV-1 using IFA. the results indicated that rabbit pAb reacted positively only with BHV-1-infected cells and exhibited no cross-reactivity with BVDV (**Figure 3C**). These results suggested that rabbit pAb had good specificity for antigen or pathogen identification.

### 3.4 Establishment and optimization of iELISA based on gD protein

**Figure 4:**
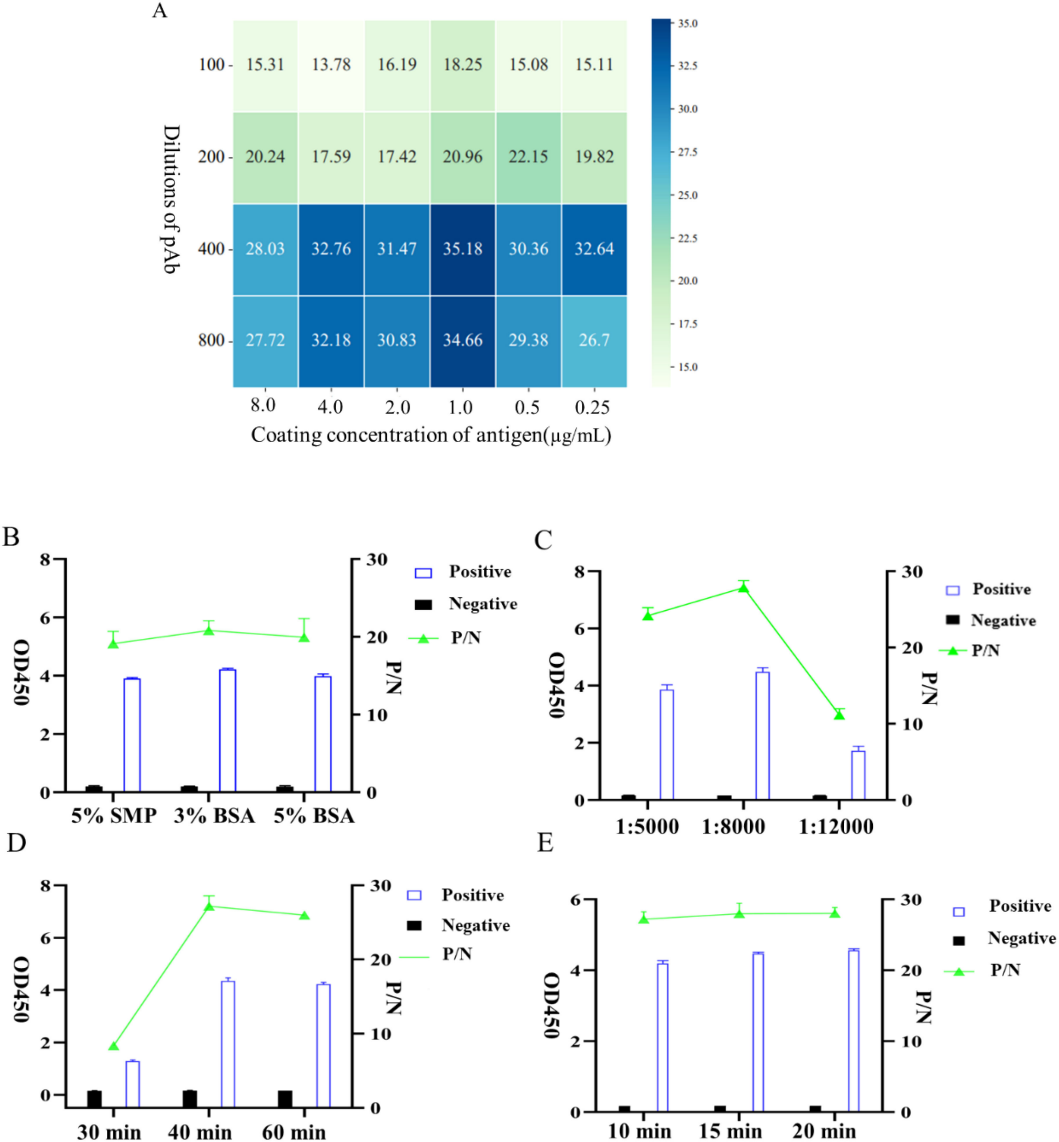
Optimization of iELISA conditions based on gD protein. **(A)** Optimal working concentrations of coated antigen and rabbit pAb were determined using checkerboard titration. The OD450 ratio (P/N) of positive to negative samples is shown in the heatmap; the higher the P/N value, the darker the color. **(B)** Determination of the three types of blocking agents. **(C)** Optimization of the working dilution of HRP-conjugated Affinipure Goat Anti-Rabbit IgG. **(D)** Optimal incubation duration of HRP-conjugated Affinipure Goat Anti-Rabbit IgG. **(E)** Determination of the optimal color development time for TMB.

The iELISA method was established using recombinant gD protein as the coating antigen, and the reaction conditions were optimized. The optimal working concentration of the coated antigen and rabbit pAb was determined by checkerboard titration, and the results showed that when the concentration of gD protein was 1.0 μg/mL and the dilution of rabbit pAb was 400, the P/N value peaked at 35.18 (**Figure 4A**), thus this condition was considered as the optimal working concentration. Furthermore, we optimized other important factors influencing the iELISA, including the type of blocking agents the incubation dilution of the secondary antibody, the reaction time of the secondary antibody, and the color development duration, and the results indicated that the reaction was optimal when 3% BSA was employed as the blocking agents (**Figure 4B**), the secondary antibody dilution was 1:8000 (**Figure 4C**), the incubation time was 45 min (**Figure 4D**), and the color development time was 15 min (**Figure 4E**).

### 3.5 Determination of iELISA cutoff value

**Figure 5:**
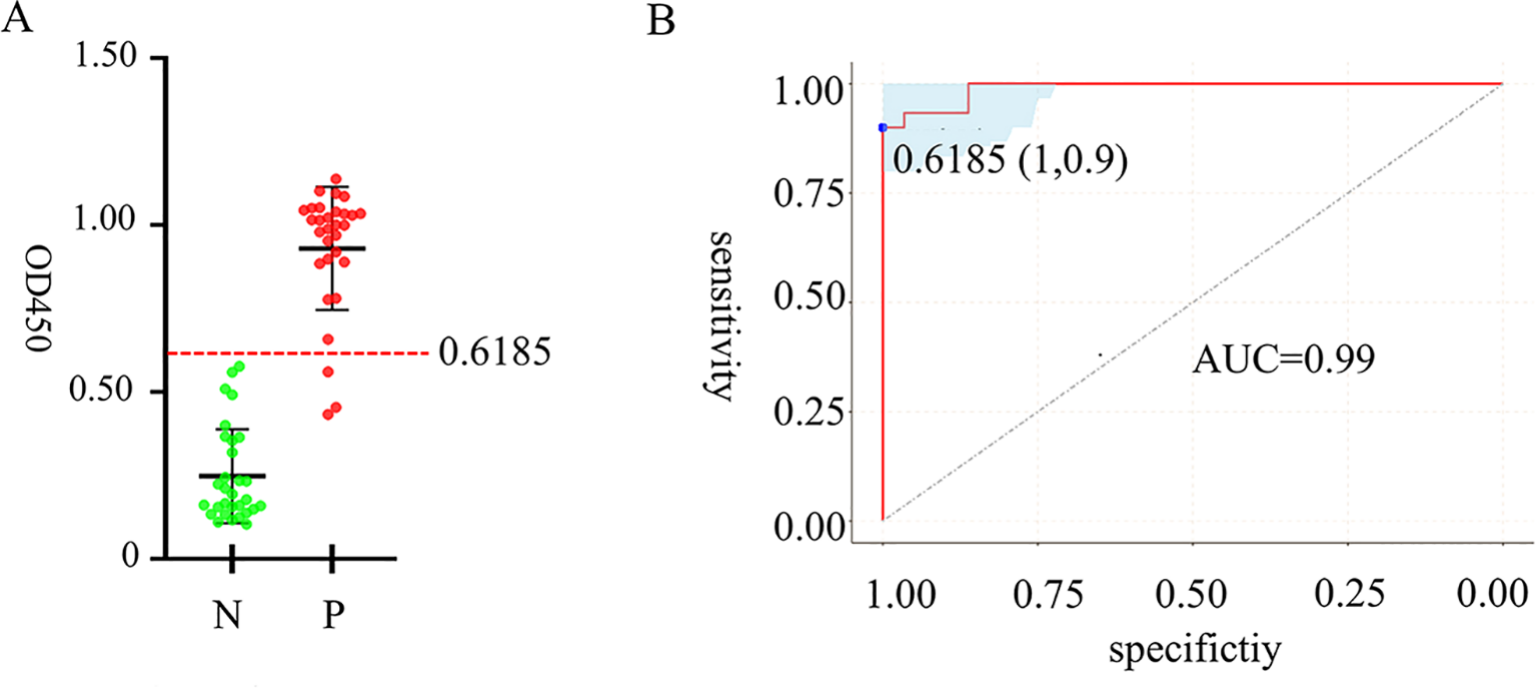
Analysis of cutoff value, detection specificity and sensitivity of iELISA based on gD protein. ROC analysis using 60 identified negative and positive clinical samples. **(A)** OD450 measurements of 60 clinical serum samples, with a red dashed line indicating the cutoff value. **(B)** ROC curve for determining cutoff value, specificity and sensitivity and AUC.

Following the optimization of the primary factors influencing the iELISA, we used 30 positive samples and 30 negative samples to assess the performance of the established iELISA (**Figure 5A**). The OD450 values of 60 samples were measured by iELISA, and statistical analysis was performed using the ROC curve, with the optimal cutoff value being 0.6185 (**Figure 5B**), at which the sensitivity of the iELISA assay was 90% and the specificity 100%, corresponding to a Yoden index of 0.9. AUC value was 0.99 (95% CI = 0.974~1), indicated a good level of accuracy for the method. According to the data obtained, we classified bovine clinical serum samples with an OD450 value beyond 0.6185 as “positive samples” and those below as negative samples.

### 3.6 Sensitivity analysis of iELISA based on gD protein

**Figure 6:**
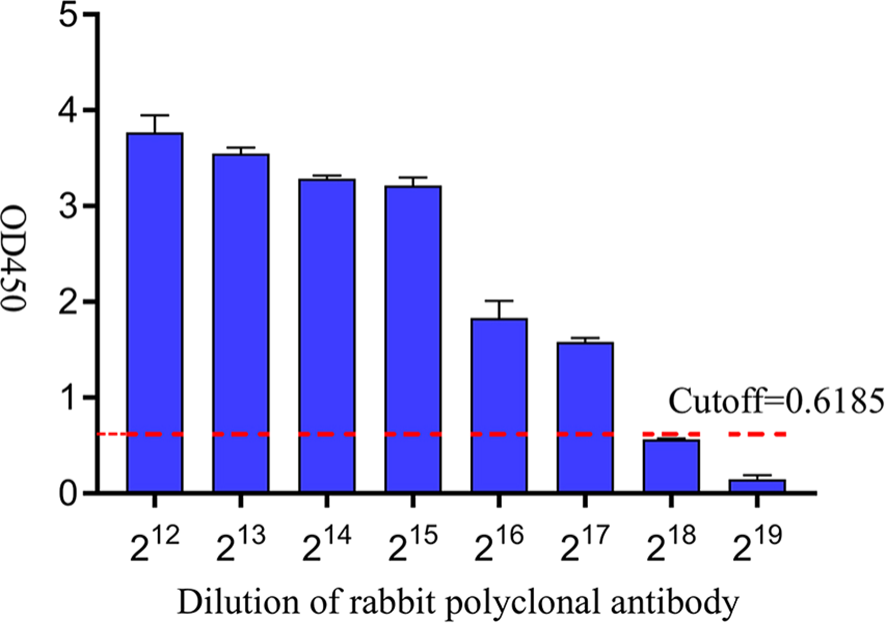
Sensitivity analysis of iELISA based on gD protein. A two-fold gradient dilution of rabbit pAb was performed on the positive samples, and the red dashed line was the cutoff value.

Rabbit pAb was serially diluted in two-fold gradient with a starting dilution of 2^12^ and positive samples were identified by the established iELISA. The results showed that the test was negative at a rabbit pAb dilution of 2^18^, indicating that the limit of the iELISA method for detecting rabbit polyclonal antibodies was 2^17^ (**Figure 6**).

### 3.7 Specificity analysis of iELISA based on gD protein

**Figure 7:**
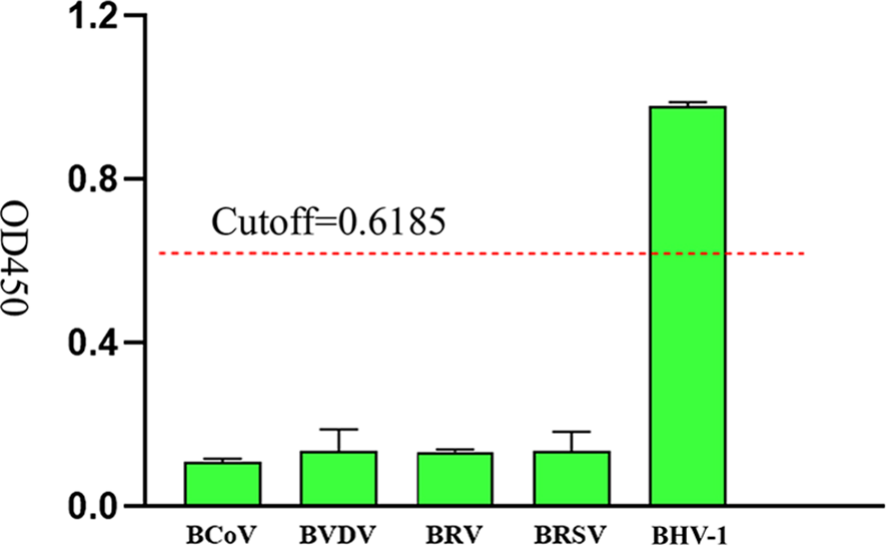
Specific analysis of iELISA based on gD protein, the OD450 values of clinical serum samples of BCoV, BVDV, BRV, BRSV, and BHV-1 were measured, and the red dashed line was the cutoff value.

To evaluate the specificity of iELISA, clinical serum samples of BCoV, BVDV, BRV, BRSV and BHV-1 bovine commonly associated viruses were tested respectively. The results of the cross-reactivity test showed that the method only exhibited good reactivity to BHV-1 samples, with OD450 values of detecting the other bovine associated viruses were all less than 0.6185 (**Figure 7**). The results showed that iELISA had no cross-reaction with other common bovine-associated viruses and had high specificity with BHV-1.

### 3.8 Repeatability analysis

In repeatability experiments, three positive serum samples and one negative serum samples were tested within a batch in single enzyme labeled plate using iELISA method, along with three positive and one negative clinical serum samples analyzed on four distinct enzyme-labeled plates. The data results showed that the intra-batch CV varied from 2.0%~6.8%, while the inter-assay CV ranged from 0.3%~4.8%, with neither exceeded 7% (**Table 2**), indicating that the iELISA exhibits good reproducibility.

**Table 2:** Repeatability analysis of the iELISA based on gD protein.

Sample		1	2	3	Average	Standard deviation	Coefficients of variability%(CV%)
Positive 1(P1)	Intra-batch	1.106	1.153	1.007	1.089	0.075	6.9
Positive 2(P2)		1.134	1.179	1.103	1.139	0.038	3.4
Positive 3(P3)		1.076	1.043	1.046	1.055	0.018	1.7
Negative 1(N1)		0.134	0.139	0.126	0.133	0.007	5.3
Positive 4(P4)	Inter-batch	1.064	1.060	1.048	1.057	0.010	0.9
Positive 5(P5)		1.079	1.074	1.105	1.086	0.017	1.5
Positive 6(P6)		1.003	1.005	1.009	1.006	0.003	0.3
Negative 2(N2)		0.156	0.151	0.142	0.150	0.007	4.8

### 3.9 Application of the developed iELISA method in clinical samples

60 bovine serum samples were tested using the developed iELISA and commercial antibody detection iELISA kits and the results were compared. four BHV-1 positive samples were detected by the commercial kit, while eight positive samples were detected by the iELISA. The diagnostic sensitivity and specificity of the iELISA method were 100% and 93.3%, respectively, with a concordance rate of 93.3% between the two methods (**Table 3**), indicating a strong agreement between the two methods.

**Table 3:** Tests of clinical Serum samples.

Samples	Commercial ELISA	Developed iELISA	Sensitivity (%)	Specificity (%)	Concordance rate (%)
Positive	4	8	100 (4/4)	93.3(56/60)	93.3 (56/60)
Negative	56	52			

## 4 Discussion

BHV-1 is highly contagious and latent; animals with BHV-1 are often ignored due to their overall good health, rendering them potential lifelong carriers of the disease (Hostnik et al., 2021). Under stressful conditions, the latent virus reactivates and disseminates to immunocompromised or vulnerable hosts, a trait that enables the virus to endure for extended durations, leading to significant economic loss to the cattle industry (Jones, 2019). Vaccination can diminish the viral load in sick animal hosts, but it can’t eliminate the virus, resulting in lifelong infection in diseased cattle, hence complicating the prevention and control of BHV-1 (El-Mayet and Jones, 2024). Efficient and precise detection technologies significantly aid vaccination efforts and the progressive development of scientific control strategies, along with the prompt monitoring and management of BHV-1 infection prevalence to ensure livestock health and production stability (Rimayanti et al., 2024).

Owing to its cost-effectiveness, rapidity, accuracy and sensitivity, ELISA has gradually become the mainstream direction of diagnostic testing for animal disease. They rely on the specific binding of antibodies to target antigens for detection, and facilitate the epidemiological investigation and research of BHV-1 by evaluating the kinetic aspects of antibody-pathogen interactions (Wang and Pang, 2024). The gD protein is considered to be one of the main structural proteins of BHV-1 infected cells. It is necessary for viral replication and can often induce more neutralizing antibodies. Due to its immunodominance and sequence conservation, it is considered to be an ideal antigen for BHV-1 virus detection and diagnosis and vaccine immune evaluation (Lewis et al., 1999). Therefore, we chose gD protein as the target for the development of iELISA method. In this study, soluble recombinant gD protein was expressed *in vitro* by a prokaryotic expression system. The system can express the target protein quickly, simply and in large quantities, and can be commercially produced at a low cost. However, since the gD protein is a highly glycosylated protein on the surface of the BHV-1 envelope, the prokaryotic expression system cannot glycosylate the protein, making it different from the natural conformation of the real protein. This reduces the conformational epitopes of the antigen to a certain extent, and reduces the diversity of the corresponding antibodies, which may affect the detection performance of iELISA. Recent literature has shown that eukaryotic expression of Sphingomyelin-activating protein-like protein 2 (SAP-2) has higher sensitivity than prokaryotic SAP-2 protein in detecting antibodies to bovine liver fluke disease, and the positive serum detection rate increased from 73% to 79.4% (Wu et al., 2025). This suggests that the eukaryotic expression system can enhance its antigenic properties and improve diagnostic accuracy, and is a promising platform for the production of specific candidate antigens for ELISA kits.

We established an iELISA based on gD protein to detect BHV-1 antibodies in bovine clinical serum samples and optimized the reaction conditions. In this study, 60 bovine clinical serum samples were tested by the iELISA, Statistical analysis of the ROC curve determined an optimal cutoff value of 0.6185 for distinguishing negativity from positivity. The AUC value was 0.99, indicating the reliability of the established mode. Youden index was defined based on all points of the ROC curve, and the optimal threshold was determined when Youden index was 0.9. When we tested several viruses commonly associated with cattle, we found that the iELISA method had no cross-reactivity with samples of BCoV, BVDV, BRV, and BRSV, and exhibited a great specificity. When performing sensitivity analysis, it was found that the minimum limit for detecting rabbit polyclonal antibodies was 2^17^, which was comparable to the sensitivity of the iELISA method developed based on VP6 protein for bovine rotavirus antibody detection (Niu et al., 2024), but better than the iELISA method described in most previous studies (Hou et al., 2022) (Wang et al., 2022). The relative sensitivity and specificity of the iELISA method established in this study were 100% and 93.3%, respectively, which were better than the previously established iELISA method for gD antibody (Ratta et al., 2020). This may be because the recombinant gD protein expressed in previous literature was affected by renaturation, which affected its binding ability with the antibody. Generally, a high sensitivity in an assay correlates with lowered specificity (Varghese et al., 2023), but in the specificity experiments of this study, the OD450 of the negative samples was not approach to the cutoff value, and the sensitivity and specificity of the established iELISA method were high, which was close to the data reported in the previous literature (Nautiyal et al., 2023), indicating that the specificity was not reduced. However, the small sample number may have contributed to potential errors in the results, and further data from clinical samples is required to substantiate the relationship between sensitivity and specificity. The concordance rate of bovine clinical serum samples detected by the developed iELISA and commercially available antibody detection iELISA kits was 93.3%, indicating that the developed iELISA based on gD protein can be a useful detection tool for large-scale monitoring of the epidemiology of BHV-1 infection.

## 5 Conclusion

In summary, we used the prokaryotic system to express soluble recombinant gD protein and successfully established an iELISA method based on gD protein for detecting BHV-1 antibodies, which has high sensitivity and specificity and has no cross-reaction with other common pathogens in cattle. The establishment of this method provides strong technical support for epidemiological investigations and studies on BHV-1.

## Data Availability

The original contributions presented in the study are included in the article/**Supplementary Material**. Further inquiries can be directed to the corresponding authors.

## Supplementary material

Supplementary Figure 1 Construction and characterization of BHV-1 gD protein vector. **(A)** Transmembrane region prediction. **(B)** Signal peptide analysis. **(C)** Hydrophilicity analysis. **(D)** B-cell epitope prediction.
