# Supplementary material for: Develop an indirect ELISA utilizing gD protein to detect antibodies against bovine herpesvirus type 1
Source: Front Cell Infect Microbiol. 2025 May 8;15:1591304. doi: 10.3389/fcimb.2025.1591304 (PMC12094974; doi:10.3389/fcimb.2025.1591304)
Supplement: Supplementary Figure 1 — Construction and characterization of BHV-1 gD protein vector. (A) Transmembrane region prediction. (B) Signal peptide analysis. (C) Hydrophilicity analysis. (D) B-cell epitope prediction. [file Presentation1.pptx]

## Slide 1
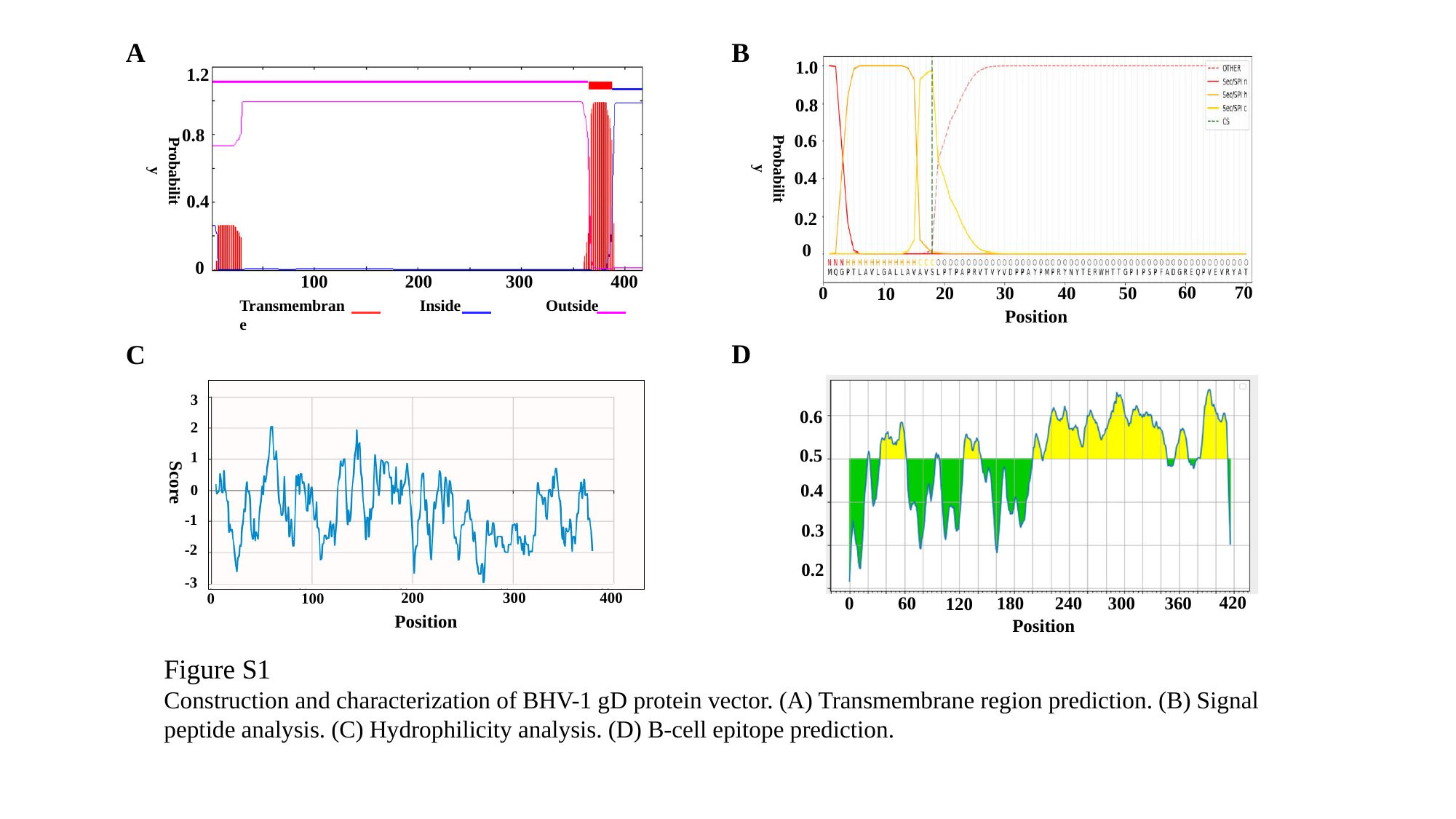

A
B
1.0
0.8
0.6
0.4
0.2
0
70
60
0
50
40
20
30
10
1.2
0.8
Probability
Probability
0.4
Transmembrane
Inside
Outside
0
100
200
300
400
Position
D
C
3
0.6
2
Score
0.5
1
0.4
0
-1
0.3
-2
0.2
-3
400
200
300
0
100
420
300
60
240
360
0
180
120
Position
Position
Figure S1
Construction and characterization of BHV-1 gD protein vector. (A) Transmembrane region prediction. (B) Signal peptide analysis. (C) Hydrophilicity analysis. (D) B-cell epitope prediction.
